# Improvement of anti-corrosion performance of an epoxy coating using hybrid UiO-66-NH_2_/carbon nanotubes nanocomposite

**DOI:** 10.1038/s41598-022-14854-y

**Published:** 2022-06-23

**Authors:** Jafar Abdi, Mazdak Izadi, Mansoor Bozorg

**Affiliations:** 1 Faculty of Chemical and Materials Engineering, Shahrood University of Technology, Shahrood, 3619995161 Iran; 2 Department of Materials Engineering, Hamedan University of Technology, Hamedan, 6516913733 Iran

**Keywords:** Chemical engineering, Materials science

## Abstract

In this study, a porous nanocontainer from UiO-66-NH_2_/CNTs nanocomposite with an excellent barrier characteristics was constructed through amine-functionalized Zr-based metal organic framework. The characterization of the prepared nano-materials were performed using different analyses such as FTIR, XRD, SEM, EDS, TEM, and BET and the results proved the successful synthesize of UiO-66-NH_2_/CNTs nanocomposite. The corrosion protection performance of the coated panels was investigated by electrochemical impedance spectroscopy (EIS), salt spray, and contact angle measurement. The EIS results revealed that unmodified and UiO-66-NH_2_ containing coating in 3.5 wt.% NaCl electrolyte were failed after 45 days but the corrosion was negligible in UiO-66-NH_2_/CNTs coating due to high pore resistance values even after 45 days. Salt spray and contact angle measurements confirmed that UiO-66-NH_2_/CNTs containing coating acts as an efficient barrier against wet saline environment even at long exposure times. This is attributed to uniform dispersion in the epoxy matrix and formation of a uniform nanocomposite coating.

## Introduction

As a promising material for delaying ions and water molecules from reaching to metals’ surface, polymeric coatings act like a physical barrier. The epoxy resins could be considered as a group of distinguished thermosetting polymers with outstanding features of excellent resistance to moisture, remarkable resistance to solvents, excellent mechanical and thermal properties, and great adhesion properties to various surfaces of both metals and non-metals. Due to its excellent properties, this material has been utilized in a wide range of industries including aircraft construction, car industries, and petroleum industries; Nevertheless, suffering from some salient weaknesses such as poor crack deflection performance, and brittleness, it couldn’t be used in many applications^1,2^. Therefore, finding a suitable alternative for epoxy resin among organic coatings is challenging as these materials are not water-proof and totally perfect. Resultantly, in a corrosive environment, metals could not be preserved for long period. Formation of defects (in micro-scales) in the structure of coatings is almost inevitable. The situation gets worse when the material is designed for outdoor application where it experiences harsh and uncontrolled conditions. Bypassing the coating material, the structural defects result in the corrosion of metal. So as to prevent such a kind of problem, a new generation of coating with the ability of anti-corrosion has been constructed. The protective ability could be improved by addition of nano/micro additives to the structure of epoxy-based coatings. Various investigations have dealt with the construction of high quality nanocomposites with excellent mechanical and thermal features^3–5^. A wide variety of nanofillers are investigated and chosen for enhancement of the coatings’ protectiveness. For example some of carbon-based nanofillers are: carbon nanotubes^6,7^, graphene and graphene oxide^8,9^, inorganic nanomaterials such as LDH^10^, fullerene^11^, and halloysite^12^ and clay^13^, etc.

In recent decades, a group of porous materials with a 3D structure have been widely investigated and developed significantly. Since introduced as materials with nanopore structure, modifiable properties by replacing its ligands, and astonishingly large surface areas, metal–organic frameworks (MOFs) have attracted many attentions^14–19^. Additionally, these porous materials have been contemplated as promising candidates for fabrication of anti-corrosion coatings with barrier capability. Furthermore, not only the resulted coatings will prevent corrosion properly, but also it has been observed that they are relatively impermeable. Unfortunately, rarely reports could be found covering the application of MOFs in the fabrication of anti-corrosion coatings or their application as a protective layer^20–31^. In this regard, there are some works that introduce bare MOFs imparted with corrosion resistance without the need of any post-synthetic modifications. Roy et al.^32^ synthesized a three dimensional supramolecular porous framework Zn(OPE-C_18_)0.2H_2_O (NMOF-1) with high water contact angles and corrosion resistance. In other study, Zhang et al.^27^ took an initiative to investigate the potential application of ZIF-8, one of the most widely studied hydrophobic and water stable MOFs, in the anticorrosion industry. Etaiw et al.^23^ obtained brown crystals of the MOF (AgCN)_4_(qox)_2_, and employed it as corrosion inhibitor for C-steel in 1 M HCl solution. Recently, Fouda et al.^33^ prepared silver based MOFs as corrosion inhibitors in acid environment and Kumaraguru et al.^29^ reported the preparation of nickel, copper, and cobalt MOFs employing the corresponding metal salts and trimesic acid as ligand and their anticorrosion properties.

Although, MOFs are extremely porous (typically have a void space of more than 40%), practical, and appropriate for many applications. The mechanical properties of the developed MOFs are yet to be understood in details. Having a plethora of outstanding features like low density, remarkable surface area, and modifiable porous structure, MOFs have been integrated into various fields. Among them, adsorption^34–37^, catalysis^38–42^, and photocatalysis^43–47^, could be mentioned. Therefore, the sophisticated composite materials could be fabricated using MOFs and their unique mechanical and thermal properties^48,49^ and anti-corrosion features^50,51^. Ma et al.^52^ added Sn-MOF@PANI and observed that the mechanical and thermal characteristics of the investigated epoxy composite coatings improved. Similarly, Zhang et al.^53^ showed that the epoxy coating’s resistance to fire was significantly intensified as a result of hybridization between MOFs and GO nanosheets.

In recent years, the thermal stability, high strength when exposed to shear forces, nanosize structure, and notable surface area of zirconium-based MOFs has caught the researcher’s imagination especially for some applications like separation of various components and drug delivery^54–59^. In recent investigations, zirconium-based MOFs have been paid attention due to their high elasticity module (G)/ shear modulus (more than 13 GPa) in comparison to the other MOFs. For example, it has been found that Zr-based MOFs generally have a shear modulus value about 5–10 times of other well-known MOFs like ZIF-8. Additionally, Zr-based MOFs have a mechanically, chemically, and thermally stable structures due to numerous organic–inorganic nodes between Zr atoms and 2-ATA. Having a perfect coordination between organic and inorganic parts, the zirconium-based MOFs benefit from outstanding mechanical features and a stable structure when experiencing a shear stress^57^. Sai et al.^60^ examined the effect of Zr_6_O_4_(OH)_4_ clusters and 1,4-benzodicarboxylic acid, as the metal node and organic ligands, respectively, on the thermal stability of the synthetized MOF in fire. Likewise, Guo et al.^49^ conducted research on covering CNTs with SiO_2_@UiO-66 particles and it was found that their resistance against flames improved significantly and the epoxy coating successfully acted like an organic–inorganic inhibitor against the corrosion issue. In order to protect metals against corrosion, scientists have synthesized new CNTs-based coatings known as the superhydrophobic coatings (SHCs)^61^. It is noteworthy to mention that the limited interactions between polymer chains and the CNTs used in the structure of epoxy and the low dispersion of CNTs in the matrix of epoxy coatings are challenging. Therefore, it is of utmost importance to control the CNTs entanglement and the uniform dispersion of them in the final coating. Surface modification of CNTs is a convenient way to reduce the interaction of nanotubes. A plethora of approaches just have focused on the enhancement of the oxygen/water/ion preventing properties of high-performance coatings which are filled with upgraded CNTs in the form of nanocomposite additive.

Considering the above concerns, composites based on the UiO-66-NH_2_ and CNTs can provide the anti-corrosion properties. Some UiO-66-NH_2_/CNTs composites have been synthesized for various applications such as fabricating transistors^62^, dye separation^63^, electrocatalytic sensing^64,65^, and photocatalytic CO_2_ reduction^66^. To the best of our knowledge, the report regarding decorating the UiO-66-NH_2_ with CNTs yet to be investigated. In the presented study, aiming for addressing the mentioned gap in the literature, authors have fabricated a UiO-66-NH_2_/CNTs nanocomposite for enabling the epoxy coating to act as an anti-corrosion coating. So as to accomplish these goals, a Zr-based MOF functionalized by amine group was employed. The properties of the developed MOF/CNTs were analyzed by various methods, such as FTIR, XRD, SEM, EDS, TEM and BET. Afterwards, electrochemical impedance spectroscopy (EIS) tests, salt spray and contact angle measurement were applied on CNTs, UiO-66-NH_2_, and UiO-66-NH_2_/CNTs incorporated epoxy composites to analyze their performance in their anti-corrosion characteristics.

## Experimental

### Materials

Zirconium tetrachloride (ZrCl_4_, 98%) and 2-Aminoterephthalic acid (ATA, 99%) were used as metal and organic precursors of MOF, respectively bought from Sigma-Aldrich. Utilized solvents including methanol, N, N-dimethylformamide (DMF, 99%), nitric acid (HNO_3_, 68%), sulfuric acid (H_2_SO_4_, 98%) and acetic acid (HAc, 98%) were provided by Merck company. Carbon nanotubes were purchased from PlasmaChem GmbH and used without further purification. Epoxy resin (Araldite GZ7 7071X75: solid content: 74–76%, epoxy value: 0.15–0.17, density: 1.08 g/cm^3^) was purchased from Saman Co. Furthermore, amido polyamide curing agent based on CRAYAMID 115 (Arkema Co.) was employed as curing agent. In this research work, mild steel (CK10) was used as substrate. The chemical composition (wt.%) of the steel was as follows: 0.1 wt.% C, 0.45 wt.% Mn, ma × 0.4 wt.% Si, and about 99 wt.% Fe. Steel panels were cut into the size of 30 × 20 × 1 mm and 80 × 50 × 1 mm. Then the surface was polished with SiC paper of graded grit sizes ranging from 120 to 1500 to achieve a mirror-shinning surface then ultrasonically cleaned in a mixture of acetone and ethanol for 10 min. Finally samples were washed with distilled water and then dried in air.

### Preparation of UiO-66-NH_2_

Primarily, zirconium tetrachloride (0.095 g) and amino-functionalized ligand, NH_2_-BDC (0.067 g) were separately dissolved in 15 mL of DMF solvent for 15 min using ultrasonic vibration. Next, the Zr^4+^ ions solution was mixed with the ligand solution containing 2 mL of acetic acid. Then, the final mixture was agitated for an hour, subsequently was moved into an autoclave (Teflon-lined stainless steel) and was heated at 120 °C in the oven during 24 h. Finally, the mixture was cooled down, centrifuged and washed several times with methanol and dried at 70 °C to obtain UiO-66-NH_2_ powder.

### Preparation of UiO-66-NH_2_/CNTs nanoparticles

To prepare hybrid UiO-66-NH_2_/CNTs nanocomposite, carbon nanotubes walls were primarily modified with negatively charged carboxyl groups (COOH^−^). This act was performed by dispersing CNTs powder (1 g) into the mixture of sulfuric acid and nitric acid (100 mL) solution using ultrasonic instrument followed by refluxing at 80 °C overnight. Then, the functionalized CNTs were filtered and washed with deionized water until pH of 6 was achieved. After that, the filter cake was dried for 24 h at 70 °C for the subsequent procedure. Similar to the mentioned method for synthesizing UiO-66-NH_2_ crystals, hybrid UiO-66-NH_2_/CNTs nanocomposite was prepared. Therefore, modified CNTs were dispersed into the DMF solution (15 mL) containing zirconium chloride and 2-Aminoterephthalic acid ligand agitated for an hour. Then, the final mixtures was transferred into stainless steel autoclave and heated at 120 °C for 24 h. Finally, the mixture was cooled down, centrifuged and washed several times with methanol and dried at 70 °C to obtain UiO-66-NH_2_/CNTs nanocomposite for further experimental tests. Fig. S1 shows a schematic of the synthesis process of UiO-66-NH_2_@CNTs hybrid nanocomposite.

### Preparation of CNTs, UiO-66-NH_2_ and UiO-66-NH_2_/CNTs epoxy nanocomposites

To investigate the effect of synthesized composite material on the electrochemical behavior of epoxy coating, 0.5 wt.% of each materials were distributed in the epoxy resin and polyamide hardener mixture (at a ratio of 5/2 w/w) through an ultrasound-assisted procedure. The epoxy/polyamide mixture was diluted to reduce the viscosity. The epoxy coating with 0.5 wt.% of synthesized materials were applied on steel sheets by a film applicator. The coated samples were kept at 25 °C for 24 h and then cured at 80 °C for 1 h. The dry thickness of the coating was about 50 μm.

### Characterization and techniques

The fingerprint and structural functional groups of the materials were determined using Fourier transmitter infrared spectroscopy (FTIR, Perkin-Elmer, Spectrum One, USA). X-ray diffraction (XRD, PANalytical, Netherlands) was employed to distinguish crystal structure and the data was collected by Bruker D8 advance diffractometer utilizing Cu/Kα radiation source and voltage 40 kV, 40 mA, and 1.54056 Å. The sample was scanned between 5 and 65.95 degrees 2θ, in step sizes of 0.01° at a rate of 0.01° 2θ/second. The scanning electron microscopy (SEM) (LEO 1455VP, Oxford, UK) and transmission electron microscopy (TEM) (Zeiss-EM10C-100 kV, Germany) were run to find out the crystal size and the surface morphology of all samples. The specific surface area of the synthesized MOF and nanocomposite were identified using Brunauer–Emmett–Teller (BET) measurements (BELSORP-mini II, BEL, Osaka, Japan) by N_2_ adsorption–desorption isotherm at 77 K.

### Electrochemical impedance spectroscopy (EIS)

In order to study the electrochemical properties, a potentiostat/galvanostat (Autolab, PGSTAT 302 N) was used. Coated samples (1 cm^2^ exposed area), saturated calomel electrode (SCE) and a platinum electrode were employed as working electrode (WE), counter electrode and reference electrode, respectively. Impedance measurements were performed by an AC signal with the amplitude of 20 mV peak to peak at the open circuit potential in the range of 100 kHz to 10 mHz frequency. The coated samples were immersed in 3.5% NaCl solution and EIS measurements were performed over time. The Nova (version 1.6) and Z-View2 softwares were used to data measurements and fit the EIS data via the electrical equivalent circuits (EEC), respectively. All EIS experiments were repeated for three times under the same conditions to ensure that the results are reproducible.

### Salt spray test

Salt spray exposure was carried out on the steel samples coated with epoxy coating. The test was done on the scratched samples for 500 h in salt spray cabinet according to ASTM B117. The NaCl concentration of salt fog was 5 wt.%. All samples were scratched with a dimension of 3 cm × 2 mm, created using a surgery knife.

### Contact angle measurements

Contact angles were measured using a home-made system at room temperature to investigate the static contact angles of distilled water droplet (5 µL) on the different coating. Water droplets were carefully placed onto the surfaces of the samples and the shape of droplet was recorded by canon digital camera. The homemade apparatus has been calibrate with standard references before measurements. The contact angle was determined from the average of five measurements at various positions on the samples surface.

## Results and discussion

### Characterization of the prepared materials

**Figure 1:**
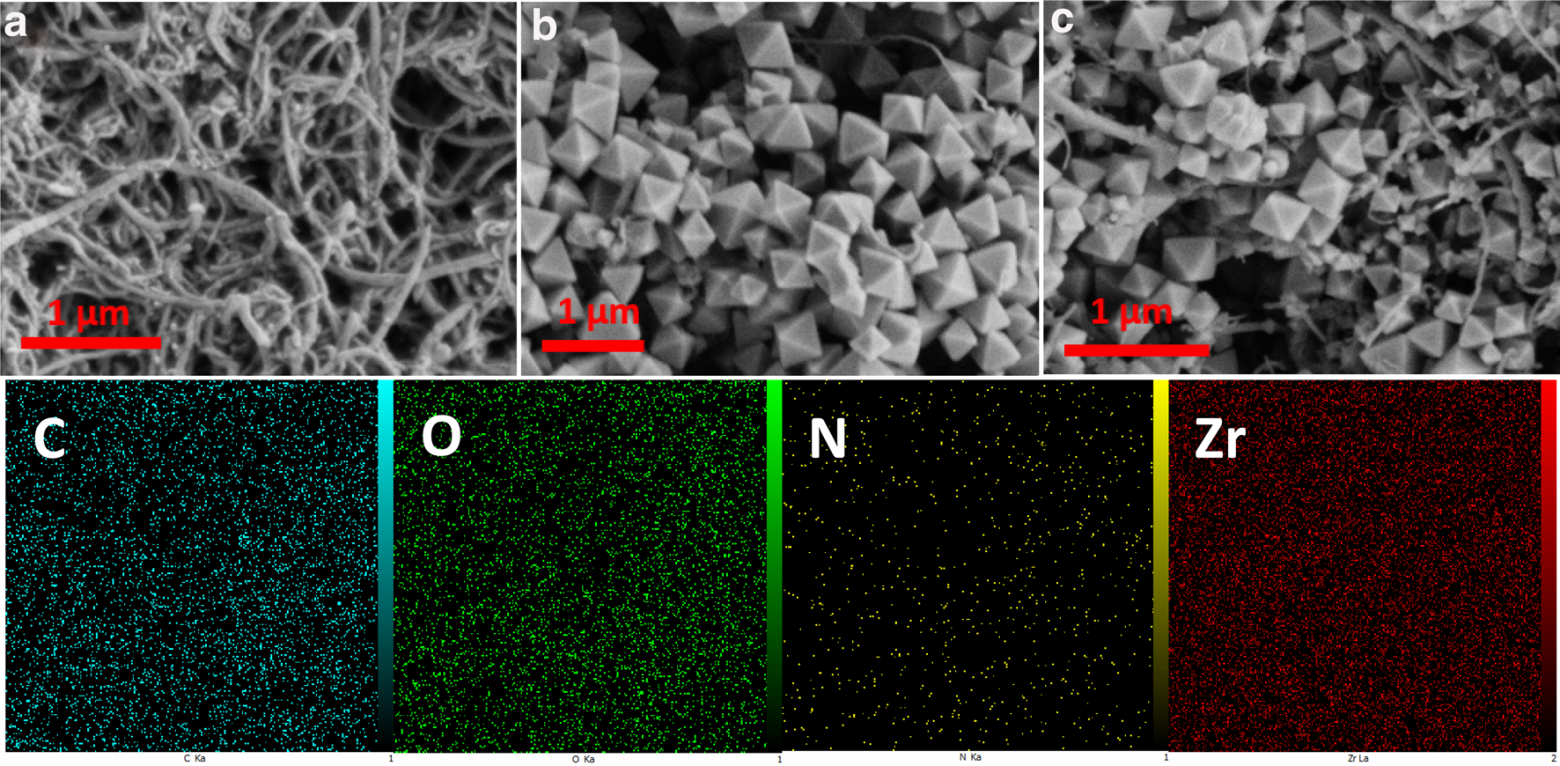
SEM images of (**a**) CNTs, (**b**) UiO-66-NH_2_, (**c**) UiO-66-NH_2_/CNTs, and EDS mapping of hybrid nanocomposite.

**Figure 2:**
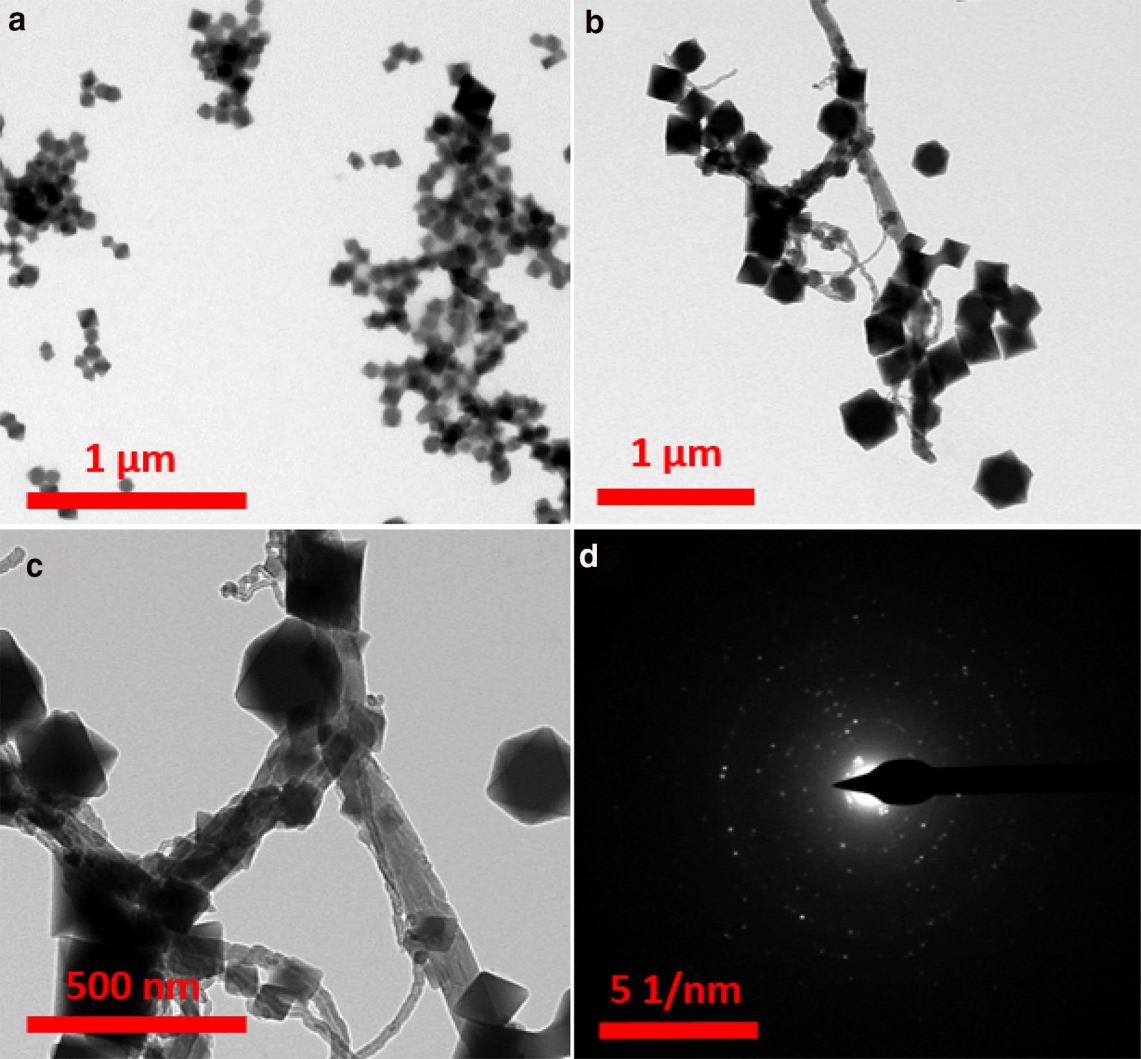
TEM images of (**a**) UiO-66-NH_2_ nanoparticles, (**b**, **c**) UiO-66-NH_2_/CNTs hybrid nanocomposite with two different magnifications, and (**d**) electron diffraction of hybrid nanocomposite.

Different characteristic analyses were investigated to confirm the successful preparation of the nanomaterials. The scanning electron microscopy (SEM) analysis was employed to evaluate the surface morphology and structural characteristics of the synthesized nanoparticles. Figure 1a shows the carbon nanotubes after acid-treatment, which possess about 10–35 nm diameter. Figure 1b illustrates the pure UiO-66-NH_2_ crystals (50–400 nm) with a uniform and octahedral shape. The morphology of hybrid UiO-66-NH_2_/CNTs nanocomposite reveals a three-dimensional interlinked network (Fig. 1c). It can be clearly seen that the CNTs and UiO-66-NH_2_ nanoparticles retain their tubular and octahedral structures after hybridization. Also, EDS mapping represents the elemental scattering of C, O, N, and Zr in the UiO-66-NH_2_/CNTs framework, verifying the successful fabrication of the nanocomposite. Further investigation was performed using transmission electron microscopy (TEM) analysis to show the micro-cavities of the nanoparticles structure. Figure 2a shows the UiO-66-NH_2_ particles, which confirms the octagonal structure with moderate aggregation. The TEM image of UiO-66-NH_2_/CNTs hybrid nanocomposite with two different magnifications, shown in Fig. 2b, c, indicates the growth of UiO-66-NH_2_ crystals on the outer wall of CNTs as clusters of grape. Also, the electron scattering pattern (Fig. 2d) for a selected area, clearly shows the bright spots in the form of a circular ring, which determines the frequency of the synthesized crystalline lattice structure. Electron diffraction is performed on a TEM by using the magnetic lenses of the beam column to focus the beam down to a point that can be aimed at a single particle or edge of a larger crystal. The result is a black image with points of light where the crystal structure is causing the beam to scatter. With the main beam blocked in the center of the image, these points of light or rings in the case of an amorphous or polycrystalline material can be used to calculate information on the crystal structure of the sample.

**Figure 3:**
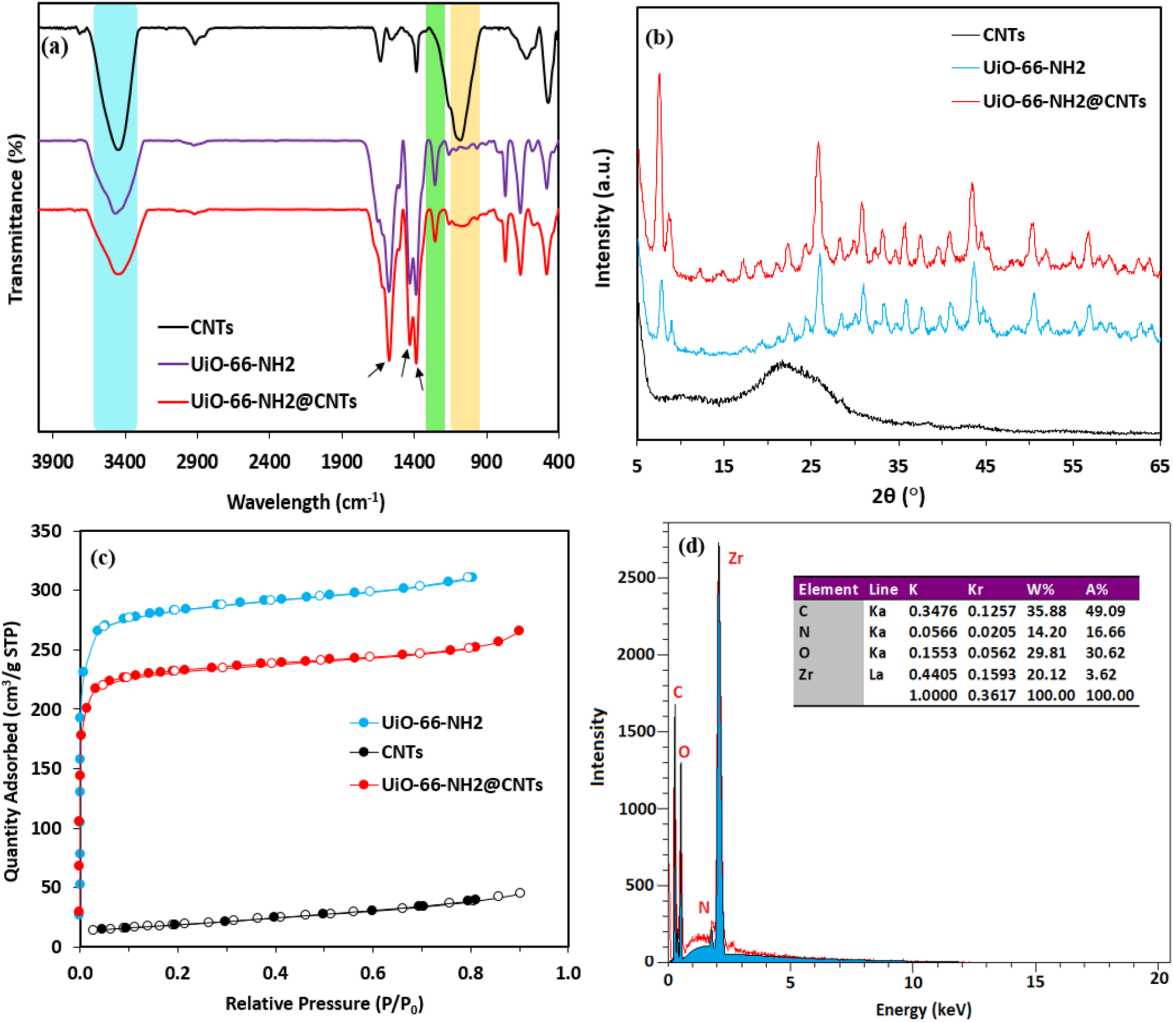
(**a**) FTIR analysis, (**b**) XRD patterns, and (**c**) N_2_ adsorption–desorption isotherms of the prepared nanomaterials at 77 K, and (**d**) EDS analysis data of UiO-66-NH_2_/CNTs nanocomposite.

The FTIR spectra of CNTs, UiO-66-NH_2_ and UiO-66-NH_2_/CNTs hybrid nanocomposite are shown in Fig. 3a. The UiO-66-NH_2_ spectrum illustrates three specific peaks (marked with small arrows) at 1400, 1580, and 1655 cm^−1^. The FTIR peak at around 1400 cm^−1^ attributed to symmetric vibration of carboxyl group. The carbonyl group vibration band appeared at 1655 cm^−1^ and the peak at 1580 cm^−1^ ascribed to aromatic C=C stretching vibration. In addition, two typical peaks located at 788 and 1260 cm^−1^ attribute to N–H and C–N stretching band and confirm the presence of –NH_2_ group in the UiO-66-NH_2_ structure. Furthermore, two characteristic peaks at 3518 and 3337 cm^−1^ are derived from the asymmetric and symmetric vibrational bands of the primary amine group, respectively^67^. Some peaks existing in the range of 400–700 cm^−1^ are belonged to in-plane and out-of-plane –COO groups^’^ vibrations^68,69^. The FTIR spectra of UiO-66-NH_2_/CNTs nanocomposite shows that the main bands are relatively remained with no variations in comparison with the pure UiO-66-NH_2_. However, some small changes can be observed at some peaks such as 574 cm^−1^, 1064 cm^−1^ (highlighted with orange color) and 1253 cm^−1^ (highlighted with green color), which is due to the interactions between CNTs and the MOF crystals (e.g. π–π interaction) and introducing functional groups brought with the acid-treated CNTs.

The phase structures of the prepared materials were further analyzed using XRD analysis for confirming the crystallinity. As shown in Fig. 3b, the characteristic peak with low intensity and a relative broad peak are observed at 23° and 43° in CNTs pattern which indicate amorphous crystal form and refer to (002) and (100) planes, respectively^70^. The XRD pattern of the UiO-66-NH_2_ sample shows a great agreement with the patterns reported in literature (Fig. S2)^68,71^. The specific peaks appeared at 2θ = 7.3°, 8.5° and 25.6° attribute to the (110), (200), and (600) planes, respectively^65^. According to the XRD pattern obtained for the UiO-66-NH_2_/CNTs nanocomposite, quite similar peaks to that of the UiO-66-NH_2_ can be seen with no significant displacement, confirming that the UiO-66-NH_2_ nanoparticles have been successfully loaded on the CNTs by employing the solvothermal preparation method.

N_2_ adsorption–desorption isotherms of the nanomaterials were also investigated to measure the BET surface area, as shown in Fig. 3c. The obtained specific surface areas comprise 65.35, 1113, and 1064.4 m^2^/g for CNTs, UiO-66-NH_2_, and UiO-66-NH_2_/CNTs, respectively (Table S1). All samples possess the type H_3_ hysteresis loop and reveal type I isotherm according to the IUPAC suggesting microporous structure of the materials (Fig. S3). Additionally, the pore size distribution of different samples were shown in Fig. S4. The uniform pore structure confirmed that the samples had well-defined mesopores. Beside the SEM analysis, the elemental composition of the UiO-66-NH_2_/CNTs sample was identified using energy dispersive spectroscopy (EDS) technique. Figure 3d represents the result of the EDS analysis which indicates the presence of carbon, zirconium, oxygen and nitrogen as the most dominant elements in the sample.

### Electrochemical studies

The EIS technique was used to study the effectiveness of synthesized nanocomposite additive on the epoxy coating life-time applied on the steel plates. Four prepared samples (See “Characterization and techniques” section) were exposed to the experimental electrolyte and EIS data were extracted in a time-dependence manner to pursue the key changes in the performance of additive-free and composite coatings over time. The electrolyte diffusion is occurred through the defects and low cross-linked regions into the organic coatings. When the electrolyte reach to the steel substrate corrosion will initiates and spreads in the coating-metal interface. So, the system degradation is inevitable at prolonged immersion times.

**Figure 4:**
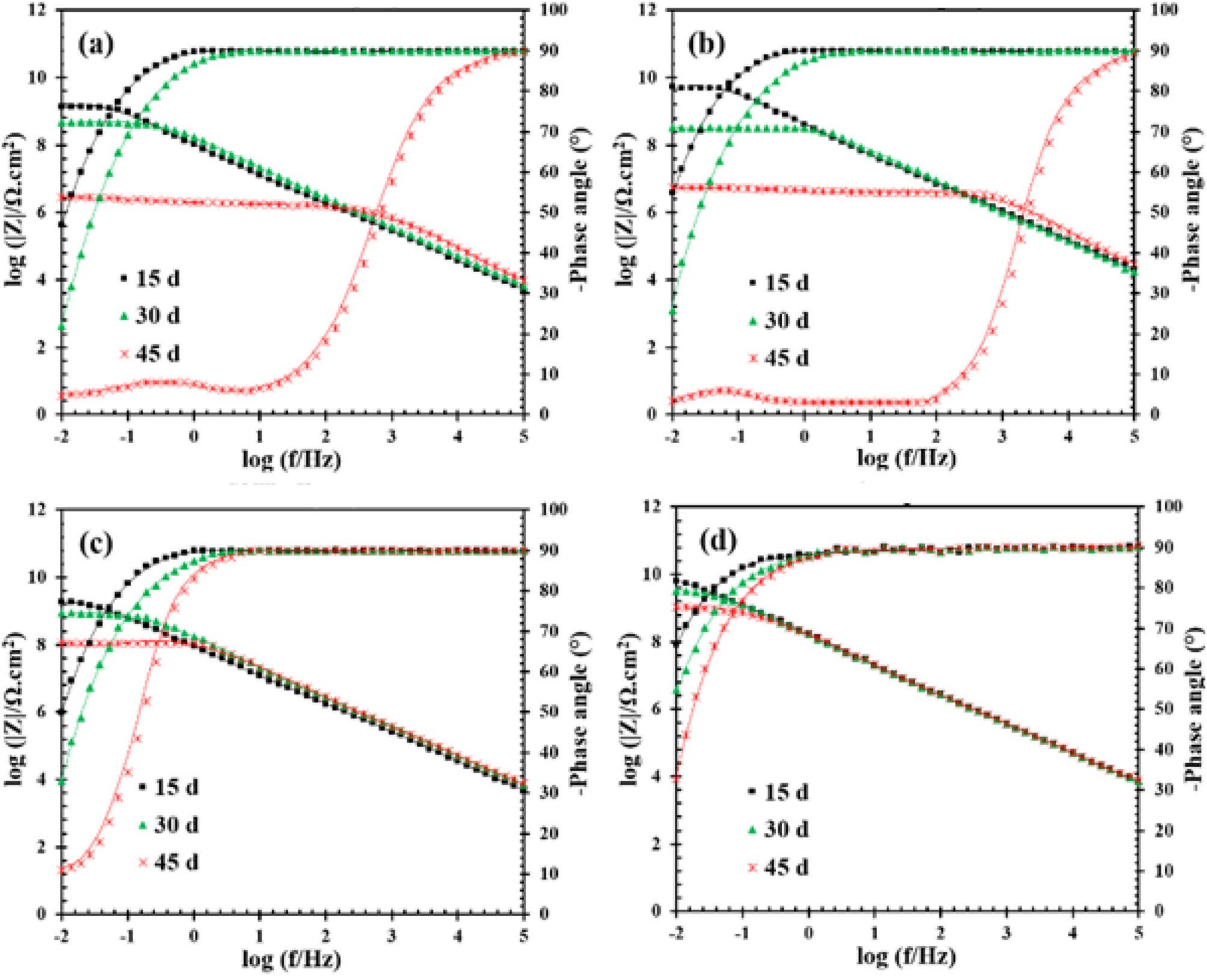
Bode plots of (**a**) unmodified/epoxy, (**b**) UiO-66-NH_2_/epoxy, (**c**) CNTs/epoxy, and (**d**) composite (UiO-66-NH_2_/CNTs/epoxy) samples after 15, 30, and 45 days at room temperature. The lines related to the fitted data.

**Figure 5:**
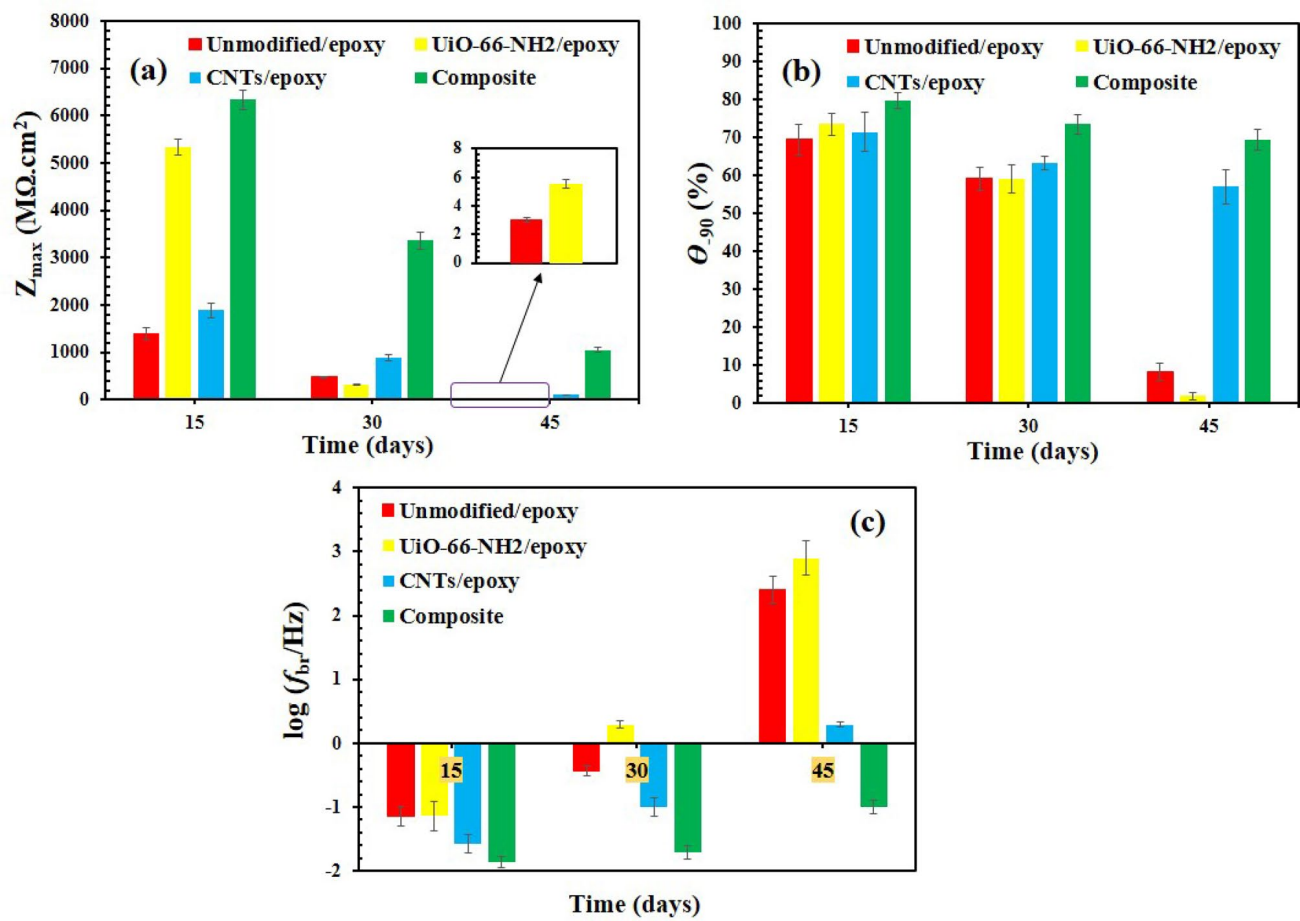
Extracted EIS parameters: (**a**) maximum impedance magnitude (*Z*_max_), (**b**) the part of Bode phase plot that is in the maximum value (θ_-90_), (**c**) breakpoint frequency (*f*_br_) at different times: 15, 30, and 45 days (All tests have been repeated three times and the corresponding error bars are illustrated in the figures).

The Bode plots, as the most beneficial output of EIS technique, are reported in Fig. 4. The key factors in the EIS study of protective organic coatings are maximum impedance magnitude (*Z*_max_), the part of Bode phase plot that is in the maximum value (θ_-90_), number of time-constants (*N*_tc_), and breakpoint frequency (*f*_br_). These factors (except *N*_tc_) are depicted in Fig. 5. As can be seen in Fig. 4, all samples showed one time-constant except unmodified/epoxy and UiO-66-NH_2_/epoxy samples after 45 days. The appearance of second time-constant could be a sign of double-layer formation in the organic–inorganic interaction face^72^. The double-layer creation is due to water uptake and consequent electrolyte accumulation in some areas of the metal-coating interface. So, the corrosion process initiated in the unmodified-epoxy and UiO-66-NH_2_/epoxy samples with longer exposures (45 days). However, the corrosion rate was not severe (See *θ*_-90_ description). Samples with one time-constant were in the immune region, although the corrosion rate could not be assumed to be zero. The resistance behavior observed in the bode-module plots of all samples showed that water penetrates to the metal surface through diffusion paths but the corrosion was negligible in CNTs/epoxy and composite samples due to high pore resistance values even after 45 days (See *Z*_max_ description). So, *N*_tc_ values and resistive-capacitance behavior of EIS plots confirmed that CNTs/epoxy and composite samples have relatively good corrosion resistance and two other samples were failed after 45 days.

As presented in Fig. 5a, the UiO-66-NH_2_/CNTs/epoxy composite and UiO-66-NH_2_/epoxy samples showed the most *Z*_max_ values after 15 days. Also, the *Z*_max_ value of UiO-66-NH_2_/epoxy is higher than the unmodified/epoxy and CNTs/epoxy after 15 days. The observed better performance is due to the better barrier performance of UiO-66-NH_2_/epoxy. The UiO-66-NH_2_ are dispersed into the coating and reinforce it against electrolyte diffusion. However, as mentioned in the introduction section, the CNTs show improper dispersion in the epoxy coating and show weaker behavior at short exposure times. However, *Z*_max_ followed a considerable decreasing trend for UiO-66-NH_2_/epoxy and reduced below 6 MΩ.cm^2^ after 45 days. Thus, organic–inorganic composite based on UiO-66-NH_2_ and epoxy were not good candidates for corrosion protection of metals without any modification. The CNTs/epoxy exhibited more acceptable protective behavior related to the unmodified/epoxy and UiO-66-NH_2_/epoxy samples at longer exposures. Although, the best performance was recorded for composite sample which *Z*_max_ was about 1000 MΩ.cm^2^ even after 45 days^73^.

According to Fig. 5b, the *θ*_-90_ values of unmodified/epoxy and UiO-66-NH_2_/epoxy samples considerably decreased over time and reached close to zero after 45 days. Despite the remarkable reduction in the *θ*_-90_ values, the phase angle at high-frequency region still was near -90 degree, indicating that unmodified/epoxy and UiO-66-NH_2_/epoxy were not fully destructed as per mentioned in *N*_tc_ description part. The recorded results for CNTs/epoxy and composite samples were different and their ascending trend was not sharp over time. The *θ*_-90_ is a good criterion for estimating the activated interface area on the metal surface^74^. The higher *θ*_-90_ values, the lower active area. So, in accordance with the *N*_tc_ results, the most *θ*_-90_ values were observed for composite sample confirming the best performance against electrolyte penetration into the interfacial face. Finally, the *f*_br_ criteria was used to peruse the coating ability in contact with high saline solution over time. *f*_br_ is an efficient criteria to evaluate the electrochemical active area in the metal/electrolyte interface^75,76^. As observed in the Fig. 5c, the lowest log *f*_br_ values were detected for composite sample at all exposure times. Also, all other samples showed positive log *f*_br_ values after 45 days and the worst results were obtained for unmodified/epoxy and UiO-66-NH_2_/epoxy. According to the obtained results, the better behavior of composite sample was proved based on all experimental criteria. If reinforcement constituent in a composite form a compatible interface with the matrix material and appropriately dispersed, the resulted compact composite coating turns to a good barrier against corrosive media.

**Figure 6:**
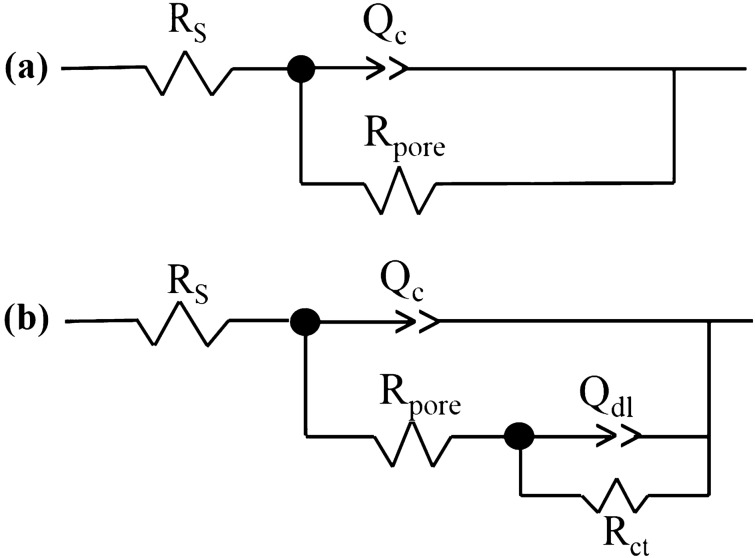
Equivalent circuit models with (**a**) one and (**b**) two time constants employed for analyzing the EIS results.

In the second part of this section, all EIS plots were fitted with appropriate equivalent circuits (ECs) and the collected data were used to better understanding the anti-corrosion behavior of composite sample. The extracted results are reported in Table 1. Also, two different ECs are illustrated in Fig. 6. The constant phase element parameter *Q* was used instead of ideal capacitor (*C*) in Fig. 6 due to the surface heterogeneity. Composite coating resistance (*R*_pore_) and constant phase element parameters (*Q*_c_ and *n*_c_) as well as double layer characteristics including charge transfer resistance (*R*_ct_) and constant phase element parameters (*Q*_dl_ and *n*_dl_) were recorded versus exposure time to find out coating-electrolyte-metal interactions. The ideal coating (*C*_c_) and double layer (*C*_dl_) capacitance were calculated as per mentioned elsewhere^77,78^.

**Table 1:** Fitting results of the prepared samples after different days extracted from EIS plots. (All tests have been repeated three times).

Sample	Time (day)	*C*_dl_ (µF)	*R*_ct_ (kΩ cm^2^)	*C*_c_ (nF)	*R*_pore_ (MΩ cm^2^)
Unmodified/epoxy	15	–	–	0.30	1399.6
	30	–	–	0.36	489.8
	45	22.76	651	0.53	2.8
UiO-66-NH_2_/epoxy	15	–	–	0.28	5236.7
	30	–	–	0.39	325.0
	45	8.35	8832	0.57	5.5
CNTs/epoxy	15	–	–	0.29	1890.0
	30	–	–	0.31	880.1
	45	–	–	0.38	104.4
Composite (UiO-66-NH_2_/CNTs/epoxy)	15	–	–	0.20	6329.6
	30	–	–	0.23	3360.0
	45	–	–	0.27	1049.2

As shown in Table 1, the relative growing trend in *C*_c_ values is detected in all samples versus immersion time. Water uptake is an inevitable phenomena in organic coating exposed to the aqueous electrolytes. So, the *C*_c_ is increased by water penetration into the composite coatings. Also, the lowest *C*_c_ values were recorded for composite sample confirming the minimum water uptake among all samples. So, although the *C*_c_ could be affected by coating swelling at prolonged exposure times, it could be concluded that the barrier action of epoxy coating was reinforced by UiO-66-NH_2_/CNTs nanocomposite additive even after relatively long exposures to high saline media^79^. However, further investigation was needed for finding the cause of lower water uptake and better corrosion protection in the composite sample. So, the hydrophobicity of the samples were studied by using of water contact angle measurement.

### Contact angle measurement

**Figure 7:**
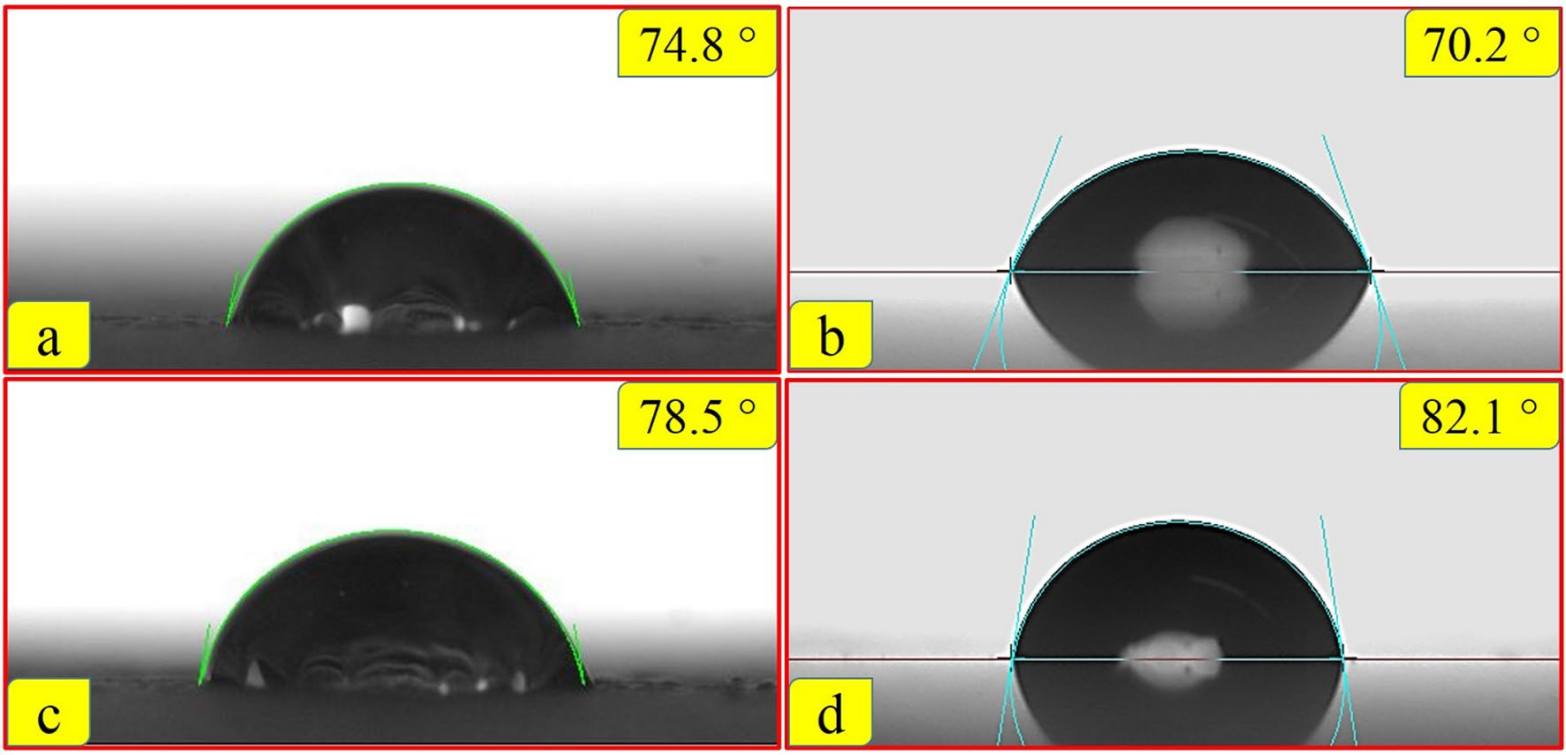
Contact angle measurements for the prepared coating samples: (**a**) pure epoxy, (**b**) UiO-66-NH_2_/epoxy, (**c**) CNTs/epoxy, and (**d**) composite (UiO-66-NH_2_/CNTs/epoxy) samples.

According to Fig. 7, the lowest contact angle values are calculated on the UiO-66-NH_2_ sample. In fact, the addition of UiO-66-NH_2_ nanoparticles to epoxy coating results in decreasing contact angle. It could be due to the hydrophilicity nature of UiO-66-NH_2_^80^. On the other hand, the composite and UiO-66-NH_2_/epoxy samples show hydrophobic characteristics and a larger water contact angle^81^. So, it seems reasonable to accept that the hydrophobicity nature of composite effectively decreased the adsorption of water onto the surface; and composite and CNTs/epoxy coatings show good barrier properties in wet environments. However, the protection behavior of composite sample is considerably better than CNTs/epoxy, especially at longer exposures. Another factor that efficiently affects the barrier behavior of a composite coating is additive dispersion in the composite matrix. The addition of CNTs into the epoxy resin without any surface treatment is difficult due to the nanotubes aggregation^82,83^. The non-uniform dispersion of CNTs creates weak regions in the final cured epoxy system which are sensitive to wet conditions^83^. The water uptake and initiation of interfacial interactions are inevitable at prolonged exposures in the presence of these weak regions. On the other hand, UiO-66-NH_2_/CNTs additive is appropriately dispersed in the epoxy media and yield a uniform nanocomposite coating acts as efficient barrier against wet saline environment even at long exposures.

### Salt spray exposure

**Figure 8:**
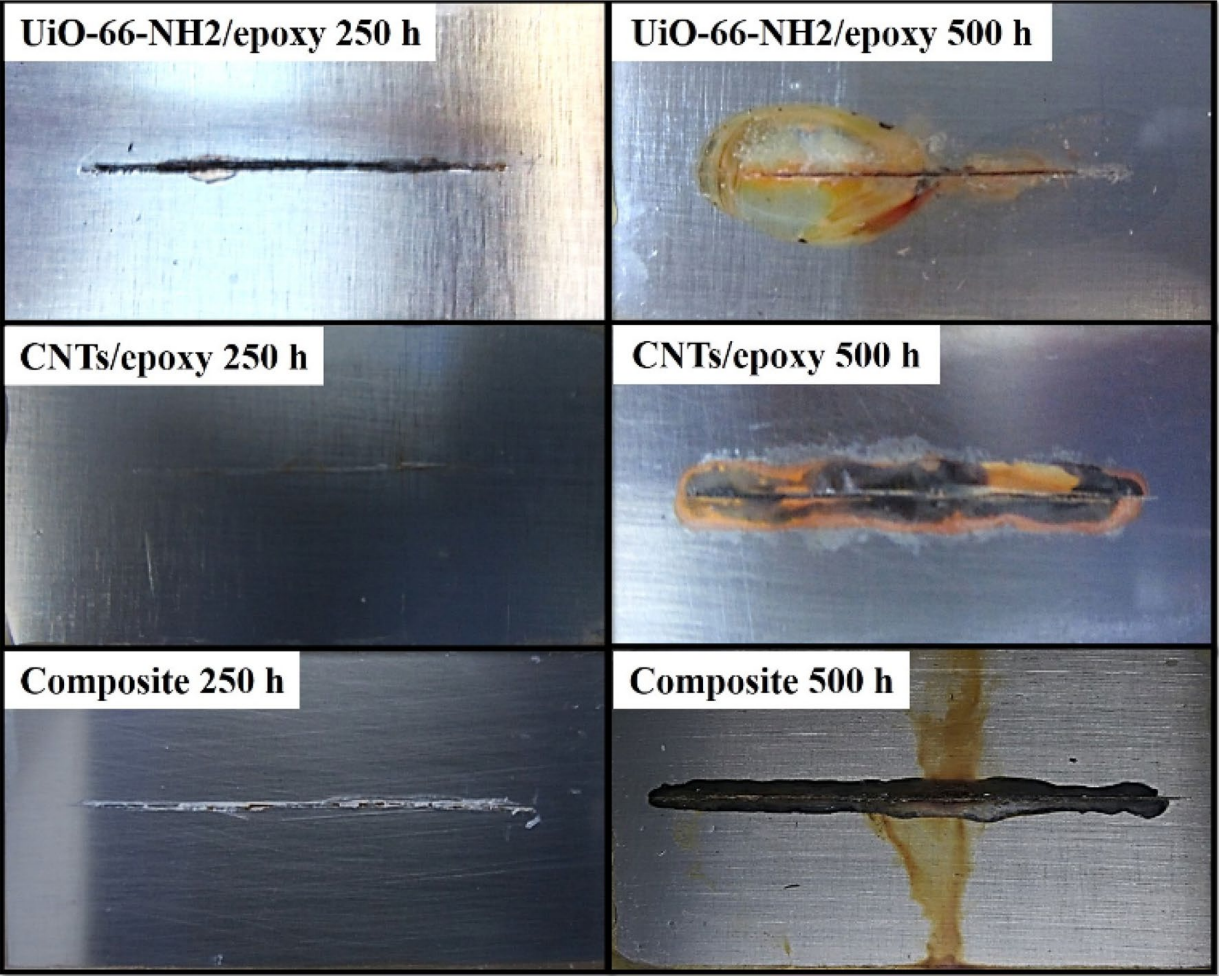
Visual performance of different epoxy coating samples with artificial scratch exposed to salt spray test over time.

The salt spray test was carried out at different exposure times on the coating with artificial defect to reveal the inhibitive performance of UiO-66-NH_2_/epoxy, CNTs/epoxy and composite samples. The visual performances of the samples are showed in Fig. 8. Corrosive media, such as chloride ions and oxygen diffuse the steel/coating interface through the micro porosities presented in the coating structure or along the scratch. When the corrosive ions penetrate the metal/coating interface, the corrosion reactions happen. So, adhesion bonds destruction and coating delamination occur due to the cathodic reactions and accumulation of corrosion product, respectively. According to Fig. 8, the corrosion reaction takes place in the scratch region of only UiO-66-NH_2_/epoxy after 250 h. By increasing exposure time up to 500 h, corrosion process is intensified and consequent products are speared around the scratch on all samples. Also, UiO-66-NH_2_/epoxy is the only sample shows coating delamination after 500 h. However, blisters are not appeared in all samples. Comparing the intensity of damaged area on scribe region indicates that addition of UiO-66-NH_2_/CNTs nanocomposite to the epoxy coating caused significant decrease of the corrosion. The smaller area of corrosion damage and better visual performance of composite sample around the scratch could be attributed to the better barrier performance against salt spray. Creation of epoxy nanocomposite coating with uniform structure and less hydrophilic nature related to non-modified epoxy coating is the key.

### Protection mechanism

As presented in previous section, the protective behavior of prepared samples could be categorized into the two mechanisms. Addition of CNTs in the form of UiO-66-NH_2_/CNTs nano-additive improve their dispersion into the epoxy matrix. So, the regions contained agglomerated additives (due to the CNTs entanglement) is not formed in the coating and the electrolyte diffusion is reduced, especially at shorter immersion times. This behavior is also observed in the UiO-66-NH_2_/epoxy after 15 days exposure due to the relatively good dispersion of UiO-66-NH_2_ additive. Another mechanism is based on the coating hydrophilicity. The water uptake and consequent corrosion of the substrate is proportional to the coating hydrophilic behavior. Despite acceptable anti-corrosion performance of the UiO-66-NH_2_/epoxy at short exposure times, its protectiveness shows considerable reduction after 30 days. However, other samples show lower reduction in *Z*_max_ value between 15 and 30 days. On the other hand, composite sample with the most contact angle provide the best protective behavior, even after 45 days. So, it can be concluded that lower hydrophilicity and more compactness are the two reasons for better corrosion protection action of composite coating in the presence of UiO-66-NH_2_/CNTs nano-additive.

## Conclusions

Fabrication of nanocomposite coating with uniform structure and hydrophobic nature related to non-modified epoxy coating is a key solution for designing an advanced protective organic coating. Accordingly, the UiO-66-NH_2_/CNTs nanocomposite was synthesized and epoxy-based coating was performed using this nanocomposite additive. FTIR, XRD, SEM, EDS, TEM and BET characteristic analyses were performed to confirm the synthesizing of UiO-66-NH_2_/CNTs nanoparticles. The outcomes showed that the UiO-66-NH_2_/CNTs nanoparticles have been successfully synthesized. The most maximum impedance magnitude (*Z*_max_) and the part of Bode phase plot that is in the maximum value (θ_-90_) and the lowest value of breakpoint frequency (*f*_br_) for UiO-66-NH_2_/CNTs/epoxy coating after long exposures in 3.5% NaCl solution indicated better performance of composite coating. The contact angle measurements represented UiO-66-NH_2_/CNTs additive increase coating hydrophobicity related to the UiO-66-NH_2_/epoxy and CNTs/epoxy coatings. According to the salt spray exposure, the smaller area of corrosion damage and visual performance around the scribe region confirmed that the addition of UiO-66-NH_2_/CNTs nanocomposite to the epoxy coating could effectively enhance corrosion properties. So, it can be concluded that the main mechanisms for better corrosion protection behavior of the composite sample are the lower coating hydrophilic nature (leading to lower water uptake) and better additives uniformity resulting in better barrier performance.

## Supplementary Information

Supplementary Information.
