# Supplementary material for: Improvement of anti-corrosion performance of an epoxy coating using hybrid UiO-66-NH2/carbon nanotubes nanocomposite
Source: Sci Rep. 2022 Jun 23;12:10660. doi: 10.1038/s41598-022-14854-y (PMC9226116; doi:10.1038/s41598-022-14854-y)
Supplement: Supplementary file 1 — Supplementary Information. [file 41598_2022_14854_MOESM1_ESM.docx]

**Supplementary Data**

Improvement of anti-corrosion performance of an epoxy coating using hybrid UiO-66-NH_2_/carbon nanotubes nanocomposite

Jafar Abdi ^a^, Mazdak Izadi ^b^, Mansoor Bozorg ^a,*^

^a^ Faculty of Chemical and Materials Engineering, Shahrood University of Technology, 3619995161 Shahrood, Iran

^b^ Department of Materials Engineering, Hamedan University of Technology, 6516913733 Hamedan, Iran

* Corresponding author. Tel.: +98-9124217836

Email: m.bozorg@shahroodut.ac.ir

| Table S1. Porosity properties of the prepared nanomaterials. | | | |
| --- | --- | --- | --- |
| Sample | Mean pore diameter (nm) | Volume pore  (P/P_o_=0.9), (cm^3^/g) | Surface area (m^2^/g) |
| CNTs | 4.23 | 0.069 | 65.35 |
| UiO-66-NH_2_ | 1.85 | 0.516 | 1113 |
| UiO-66-NH_2_@CNTs | 1.73 | 0.451 | 1046.6 |


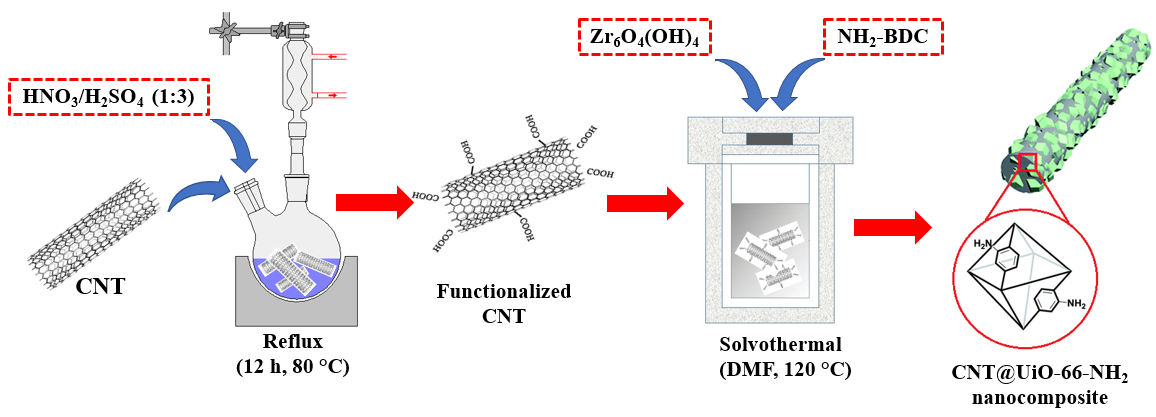


Fig. S1. Schematic of the synthesis process of UiO-66-NH_2_@CNTs hybrid nanocomposite ^1^.


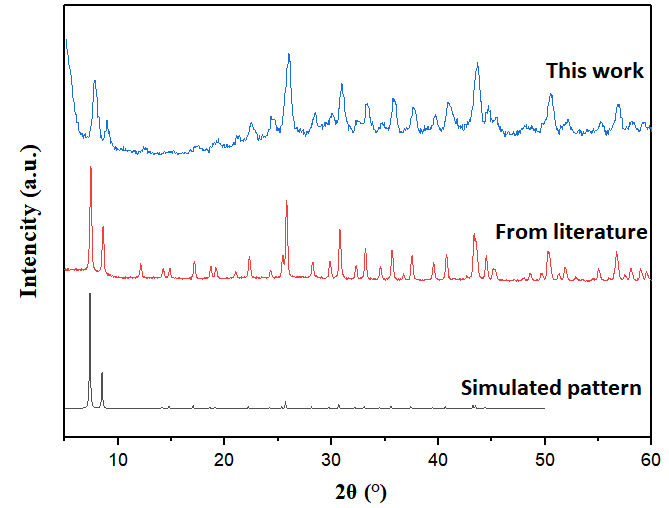


Fig. S2. The XRD pattern of bare UiO-66-NH_2_ compared with the literature ^1^.

**CNTs**

**UiO-66-NH_2_**

**UiO-66-NH_2_@CNTs**

Fig. S3. Magnified images of the N_2_ adsorption-desorption isotherms of the prepared samples at 77 K.


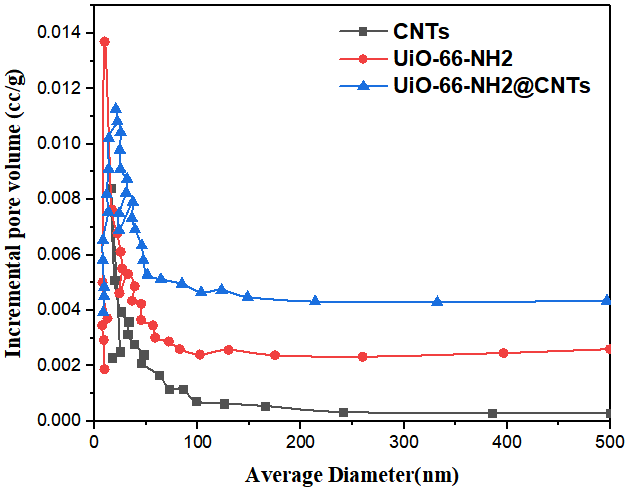


Fig. S4. Pore size distribution of the different samples.

1 Abdi, J., Banisharif, F. & Khataee, A. Amine-functionalized Zr-MOF/CNTs nanocomposite as an efficient and reusable photocatalyst for removing organic contaminants. *Journal of Molecular Liquids*, 116129 (2021).
